# DFN-YOLO: Detecting Narrowband Signals in Broadband Spectrum

**DOI:** 10.3390/s25134206

**Published:** 2025-07-05

**Authors:** Kun Jiang, Kexiao Peng, Yuan Feng, Xia Guo, Zuping Tang

**Affiliations:** 1 National Key Laboratory of Intelligent Spatial Information, Beijing 100029, China; jiangkunzzy@126.com (K.J.); m202373078@hust.edu.cn (K.P.); fengyuan990615@gmail.com (Y.F.); guoxia9407@sina.com (X.G.); 2 Beijing Institute of Tracking and Telecommunications Technology, Beijing 100094, China; 3 School of Electronic Information and Communications, Huazhong University of Science and Technology, Wuhan 430074, China

**Keywords:** signal detection, deformable channel feature fusion network, Focal_SIoU

## Abstract

With the rapid development of wireless communication technologies and the increasing demand for efficient spectrum utilization, broadband spectrum sensing has become critical in both civilian and military fields. Detecting narrowband signals under broadband environments, especially under low-signal-to-noise-ratio (SNR) conditions, poses significant challenges due to the complexity of time–frequency features and noise interference. To this end, this study presents a signal detection model named deformable feature-enhanced network–You Only Look Once (DFN-YOLO), specifically designed for blind signal detection in broadband scenarios. The DFN-YOLO model incorporates a deformable channel feature fusion network (DCFFN), replacing the concatenate-to-fusion (C2f) module to enhance the extraction and integration of channel features. The deformable attention mechanism embedded in DCFFN adaptively focuses on critical signal regions, while the loss function is optimized to the focal scaled intersection over union (Focal_SIoU), improving detection accuracy under low-SNR conditions. To support this task, a signal detection dataset is constructed and utilized to evaluate the performance of DFN-YOLO. The experimental results for broadband time–frequency spectrograms demonstrate that DFN-YOLO achieves a mean average precision (mAP50–95) of 0.850, averaged over IoU thresholds ranging from 0.50 to 0.95 with a step of 0.05, significantly outperforming mainstream object detection models such as YOLOv8, which serves as the benchmark baseline in this study. Additionally, the model maintains an average time estimation error within $5.55\times 10^{-5}$ s and provides preliminary center frequency estimation in the broadband spectrum. These findings underscore the strong potential of DFN-YOLO for blind signal detection in broadband environments, with significant implications for both civilian and military applications.

## 1. Introduction

Traditional signal detection methods primarily rely on expert knowledge-based features and can be categorized into four main types: matched filtering, cyclostationary methods, eigenvalue detection, and energy detection (ED) [1]. Regarding matched filtering, Diamant investigated a normalized matched filter (NMF) detector for a large time–bandwidth product *N* [2]. Concerning cyclostationary methods, a recent work proposed a blind radar signal separation algorithm based on the third-order degree of cyclostationarity criteria [3]. In eigenvalue detection, joint detection and delay-Doppler estimation algorithms for MIMO radars have been developed to enhance spectrum sensing precision [4]. For energy detection, ED remains a widely used technique due to its low complexity and its non-requirement for prior signal information, and it has been applied in non-cooperative burst detection and synchronization tasks [5]. In addition, Zhang et al. [6] proposed an enhanced multidimensional harmonic retrieval technique to improve spectral parameter estimation in wireless channel sounding scenarios, demonstrating the importance of accurate frequency-domain modeling in wireless signal processing.

However, despite their popularity, traditional signal detection methods mainly rely on spectrum analysis and statistical features, and their effectiveness can be limited in complex electromagnetic environments and low-signal-to-noise-ratio (SNR) scenarios. A comprehensive review of narrowband interference mitigation techniques [7] further illustrates the limitations of conventional approaches in dynamic wireless environments, particularly in addressing weak signals and broadband interference. Moreover, recent learning-based approaches, such as the transformer-based method proposed for satellite TT&C signal detection under restricted conditions [8], show improved adaptability in low-SNR scenarios.

In contrast, signal detection methods integrated with deep learning techniques provide new avenues for tackling this challenge [9]. For instance, Hiremath et al. used time–frequency analysis to extract signal spectral content and performed feature extraction through convolutional neural networks [10]. Prasad et al. proposed a blind time–frequency localization method for wireless signals, transforming signal detection into an object detection problem by converting the time series into a spectrogram and applying a faster R-CNN-based deep learning system [11]. Chen et al. introduced a deep convolutional neural network-based method for detecting linear frequency-modulated signals, achieving high-accuracy signal recognition under low-SNR conditions by replacing traditional Fourier transform techniques [12]. Dr. Li Rundong, based on the idea of transforming signal detection into object detection, proposed an improved object detection network [13] based on You Only Look Once (YOLO) v3, a real-time object detection algorithm that directly predicts bounding boxes and classes in one forward pass, aiming to further enhance the performances of deep learning methods in signal blind detection tasks. This work demonstrated that YOLO series methods [14,15,16] outperform faster region convolutional neural networks (FRCNNs) [17,18] and traditional energy detection methods in this task. Inspired by this, Guo et al. modeled wireless radio systems to construct datasets and then improved YOLOv5 by introducing a weighted bidirectional feature pyramid network and replacing the loss function with EIoU, achieving detection and modulation recognition of signals above 0 dB in time–frequency spectrograms [19].

In the field of deep learning-based computer vision, several novel research ideas have emerged that, although not directly applied to signal detection, might enhance the accuracy and precision of signal detection when introduced into this domain. For instance, deformable convolutional networks (DCNs) [20] can better capture irregular signal features by flexibly adjusting convolution and pooling layers. Online convolutional re-parameterization (OREPA) [21] enables the online optimization of convolution parameters, which may improve the model’s adaptability in dynamic signal environments. The deformable attention (DAttention) mechanism [22] can enhance signal extraction accuracy by adaptively focusing on critical signal regions. Additionally, dynamic snake convolution [23], based on topological geometric constraints, effectively segments local structures within signals and can be combined with deformable convolutions. SCConv [24] alleviates feature redundancy via spatial and channel reconfiguration, improving detection efficiency. Context-guided spatial feature reconstruction [25] leverages contextual information to optimize spatial features, thereby enhancing signal perception. Deformable large-kernel attention (DLKA) [26], combining the broad receptive field of large convolution kernels and the flexibility of deformable convolutions, improves the detection of small and irregular-shaped targets. Combining these methods could provide a new optimization pathway for signal detection, especially in handling low-SNR, complex backgrounds and high-dimensional signals, demonstrating potential advantages.

Despite the success of existing deep learning methods in signal detection tasks, significant challenges remain in handling low-SNR scenarios and broadband spectrum conditions. In particular, the broadband signal detection dataset constructed in this study can effectively simulate realistic electromagnetic environments such as military communication or cognitive radio systems. For example, the presence of weak, narrowband, and burst-like signals embedded in broadband noise is common in non-cooperative communication scenarios. Our dataset reflects such conditions by simulating multiple signals with varying SNRs, modulation schemes, and spectral distributions, enabling the proposed deformable feature-enhanced network–You Only Look Once (DFN-YOLO) model—composed of deformable channel feature fusion network (DCFFN) and YOLO architectures—to be validated under practical constraints. Therefore, the proposed approach holds promising potential for deployment in real-world applications where robust low-SNR signal detection is required. The proposed DFN-YOLO, an improvement based on YOLOv8n [27], addresses these challenges by introducing mechanisms that enhance the model’s ability to focus on narrowband signals and improve the detection of difficult samples under low-SNR conditions.

Compared with existing YOLO-based signal detection methods, such as the YOLOv3-based model detailed in [13] and the improved YOLOv5 approach detailed in [19], the proposed DFN-YOLO introduces two major innovations tailored specifically for broadband signal detection under low-SNR conditions. First, DFN-YOLO replaces the standard concatenate-to-fusion (C2f) module with the novel DCFFN module, which incorporates deformable attention mechanisms and multi-branch channel fusion, enabling the model to better extract sparse and irregular signal patterns in noisy broadband environments. Second, a new loss function, focal scaled intersection over union (Focal_SIoU), is designed to enhance sensitivity to weak signals and hard-to-detect targets, which are common in practical spectrum sensing tasks. These design choices distinguish DFN-YOLO not only from earlier YOLO-based models optimized for image tasks but also from prior adaptations that lacked mechanisms specifically targeted at low-SNR and broadband signal characteristics. The main contributions of this study are summarized as follows:

1. This study proposes a module named DCFFN, which employs a channel feature fusion process based on multi-branch paths and feature aggregation mechanisms. Through the deformable attention mechanism, the module flexibly adjusts the attention weights across different channel feature maps, adaptively focusing on more critical signal regions, thereby enabling more precise and efficient feature extraction.
2. To address the issues of insufficient hard sample learning and inaccurate bounding box localization in signal detection tasks, this study introduces the Focal_SIoU loss function. By dynamically adjusting the weights of hard and easily classified samples and optimizing the bounding box regression strategy, the model’s sensitivity to weak signals and small-target signals is improved, enabling it to more accurately capture the boundary information of the target signal.
3. To tackle the data scarcity problem in broadband signal detection, we propose an efficient method for constructing a broadband signal detection dataset. This method not only constructs datasets under various SNR conditions and, also, based on existing baseband signals, extends the dataset to complex broadband signal scenarios through simulation and data generation techniques.
4. To comprehensively assess the contributions of the model structure and optimization strategies, we design and conduct ablation experiments. The results clearly indicate that both the DCFFN structure and the introduction of the Focal_SIoU loss function significantly enhance the model’s detection performance. The trained DFN-YOLO model achieves a mean average precision (mAP_50–95_) of 0.850, averaged over IoU thresholds ranging from 0.50 to 0.95 with a step of 0.05, with the signal time estimation error controlled within $5.55\times 10^{-5}$ s, demonstrating exceptional detection accuracy and time estimation capability, outperforming existing mainstream methods.

## 2. Related Studies

In the field of wireless communication, with the continuous development of deep learning technologies, many deep learning-based signal detection methods have gradually been proposed and applied to practical problems. Compared with traditional methods, deep learning can automatically learn complex features from data, eliminating the need for cumbersome manual feature design, and it has shown stronger robustness and accuracy in handling complex channels and dynamic environments.

However, despite the significant progress that deep learning has made in many scenarios, signal detection under low-SNR and broadband spectrum conditions still faces significant challenges.

For instance, for low-SNR scenarios, Ke et al. proposed a deep learning method that combines CNN and LSTM, which can achieve efficient signal detection with little or no prior information, showing an improvement of approximately 4.5–5.5 dB over traditional blind detection algorithms [28]. A deep spectrum sensing approach based on a CNN–Transformer hybrid architecture was introduced to enhance feature interaction between the time and frequency domains, showing strong robustness under channel variation and low-SNR conditions without relying on prior channel knowledge [29].

In the field of broadband spectrum detection, Huang et al. proposed a power spectral subcarrier signal detection method based on fully convolutional networks (FCNs), treating the broadband spectrum as a one-dimensional image and distinguishing subcarriers from noise using deep convolutional neural networks [30]. Li et al. developed a method to distinguish underwater acoustic communication signals from noise using a combination of impulse noise preprocessing, GANs, and CNNs [31]. Regarding signal feature extraction, Li et al. combined S-transform with CNNs to detect frequency-hopping signals, achieving higher detection accuracy under low-SNR conditions through normalized time–frequency feature extraction [32]. Olesiński and Piotrowski estimated the noise distribution in broadband radio spectrograms using deep learning, achieving over a 6 dB improvement by subtracting noise [33].

Other approaches focus on specific signal types or environments. Selim et al. proposed a CNN-based radar signal detection method for spectrum sharing, achieving nearly 100% detection probability at 6 dB, though performance dropped at low SNRs [34]. Ha and Song fused deep learning with multipath channel information to reduce BER to $10^{-2}$ at a 7.6 dB SNR [35]. Mendis et al. used spectral correlation features and a DBN for detection, achieving 90% accuracy above 0 dB, though computational complexity remained high [36].

In broadband spectrum scenarios, Xu et al. applied a region-based CNN and GoogLeNet for spectrum hole detection, achieving 62.16% accuracy at 6 dB, but performance decreased under low-SNR conditions [37]. Qiao et al. proposed AE-LSTM, achieving 0.4 BER at 0 dB [38]. Li et al. used keypoint estimation and multi-regression to detect and classify broadband signals with mAP50 of 96% at 0–10 dB, though performance under low-SNR conditions is still limited [39].

Some methods aim to push detection boundaries under ultra-low-SNR conditions. Seo and Nam used CNNs and image encoding to detect burst signals, achieving 20% recall at −14 dB [40]. Nuhoglu et al. combined Bi-LSTM with denoising autoencoders, achieving 75% detection at −5 dB with zero false alarms [41]. Xu et al. leveraged cyclostationary properties to detect OFDM signals, reaching 30% at −4 dB [42]. Lin et al. proposed SigdetNet using the Welch periodogram input, achieving 68% detection at −4 dB but with a high 32% false alarm rate [43].

Finally, Vagollari et al. modified YOLO for spectrogram-based object detection in broadband environments. While effective in some cases, the model’s detection stability and accuracy in low-SNR environments remain to be improved [44].

Based on the existing research, it is evident that deep learning methods show great potential in signal detection, particularly those that transform signal detection into object detection tasks. However, several limitations remain, such as insufficient exploration of signal detection below 0 dB and unsatisfactory performance under low-SNR and broadband spectrum conditions.

Therefore, this study proposes the DFN-YOLO model, which enhances detection capability for low-SNR and challenging signal environments, aiming to address the accuracy bottlenecks of signal detection under low-SNR and broadband conditions.

## 3. Proposed Framework

DFN-YOLO is an enhanced signal detection model that is proposed in this study, which incorporates the DCFFN module to improve the network structure and utilizes focal loss to optimize the loss function calculation. Integrating DCFFN, designed to handle small-target signals more effectively, significantly enhances the model’s focus adjustment capabilities, especially in complex and low-SNR environments. The focal loss function, on the other hand, addresses the class imbalance by giving more weight to hard-to-detect signals, thereby improving detection performance.

### 3.1. Deformable Channel Feature Fusion Network

**Figure 1:**
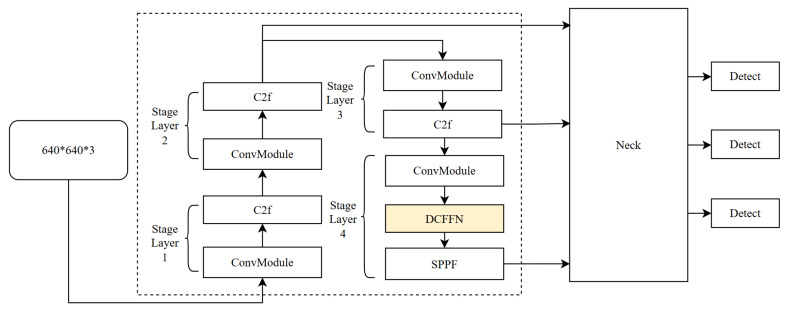
Overall structure of the proposed DFN-YOLO model, including the backbone, neck, and detection head. DCFFN replaces the original C2f module to enhance feature extraction.

The C2f structure in YOLOv8 serves as the fundamental component for multiple feature extraction layers in the backbone, improving target detection by optimizing feature extraction and information flow. However, when dealing with very small signals or signals in complex backgrounds, the performance of C2f is limited due to its simplified feature fusion approach and shallow feature expression depth. To overcome these limitations, we propose enhancing the model with the DAttention mechanism, forming the DCFFN module. This modification is specifically tailored for small-object detection, enabling the model to dynamically adjust its focus. The DAttention mechanism generates dynamic reference points that allow the model to adaptively refine its attention on key features of narrowband small-target signals. The improved DCFFN structure is applied to the orange module in Figure 1.

**Figure 2:**
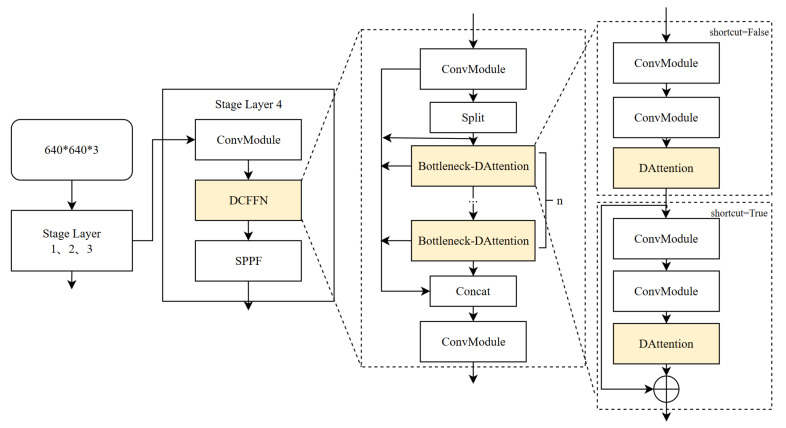
Architecture of the DCFFN backbone module. The structure integrates deformable attention and channel fusion to enhance signal region localization under low-SNR conditions.

As shown in Figure 2, DCFFN replaces the original C2f structure in the fourth layer of YOLOv8 with a DCFFN module that integrates deformable attention. The main improvement of this module lies in replacing the standard bottleneck unit with a bottleneck-DAttention unit that incorporates deformable attention. This change allows each layer to more effectively dynamically adjust and enhance the input features, particularly for small-target signals that are difficult to distinguish in complex backgrounds.

The bottleneck unit performs feature extraction and transformation through two convolutional layers, whereas the bottleneck-DAttention introduces a deformable attention layer. This layer is placed after the second convolutional layer and directly operates on the output feature map of the second convolutional layer.

**Figure 3:**
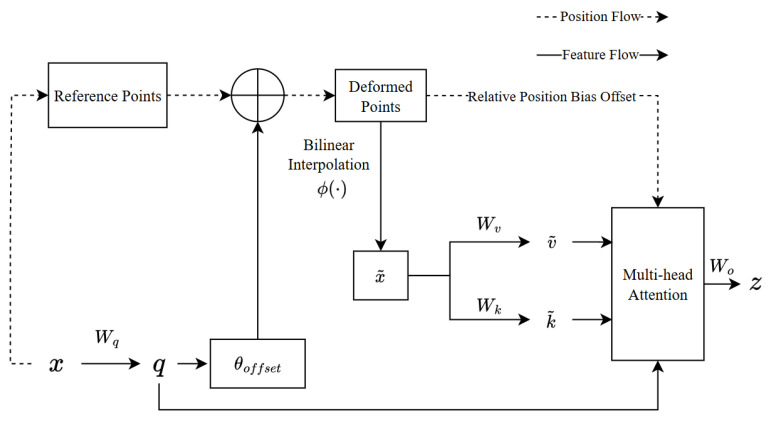
Detailed structure of the deformable attention (DAttention) mechanism used in DCFFN. The module adaptively attends to signal-relevant regions in time–frequency space. The “plus” circles in the figure indicate the element-wise addition between the reference points and the offsets to generate the deformed points.

The DAttention layer dynamically adjusts the attention focus, allowing the network to concentrate on regions of the feature map more important for the final detection task. Its structure is shown in Figure 3.

Based on the input feature map given $x\in R^{H\times W\times C}$, DAttention generates a set of reference points $p\in R^{H_{G}\times W_{G}\times 2}$. The size of the grid, $H_{G}=H/r$ and $W_{G}=W/r$, is down-sampled from the input feature map according to the down-sampling rate *r*. The values of the reference point set are the linearly spaced 2D coordinates $\left\{\left(0,0\right),\ldots ,\left(H_{G}-1,W_{G}-1\right)\right\}$, which are then normalized to [−1,+1] according to the grid shape $H_{G}\times W_{G}$, where (−1,−1) represents the top-left corner and (+1,+1) represents the bottom-right corner. To obtain the offset for each reference point, the feature map is linearly projected to obtain queries $q=xW_{q}$. The offset $\Delta p=\theta_{offset}\left(q\right)$ is then generated by passing the queries into a lightweight network $\theta_{offset}\left(\cdot \right)$. The features at the deformed points are sampled as the keys (k) and values (v) using the following projection matrix:

(1) $$q=xW_{q},\tilde{k}=\tilde{x}W_{k},\tilde{v}=\tilde{x}W_{v}$$

(2) $$\Delta p=\theta_{offset}\left(q\right),\tilde{x}=\varphi \left(x;p+\Delta p\right)$$

where $\tilde{k}$ and $\tilde{v}$ represent the deformed features of *k* and *v*. φ(·;·) represents the bilinear interpolation function as follows:

(3) $$\varphi \left(z;\left(p_{x},p_{y}\right)\right)=\sum_{\left(r_{x},r_{y}\right)}g\left(p_{x},r_{x}\right)g\left(p_{y},r_{y}\right)z\left[r_{y},r_{x},:\right]$$

where g(a,b)=max(0,1−|a−b|) and $\left(r_{x},r_{y}\right)$ index all positions on $z\in R^{H\times W\times C}$. Since g(·) is only nonzero at the four integration points closest to $\left(p_{x},p_{y}\right)$, it simplifies the weighted average of $\varphi \left(z;\left(p_{x},p_{y}\right)\right)$ to four positions in Equation (3). Similarly to existing methods, calculations of the multi-head attention mechanism are performed on q,k,v, and relative position offsets *R* are used. The output of the attention detection head is

(4) $$z^{\left(m\right)}=\sigma \left(q^{\left(m\right)}{\bar{k}}^{(m)T}/\sqrt{d}+\varphi \left(\hat{B};R\right)\right){\tilde{v}}^{\left(m\right)}$$

where $\varphi \left(\hat{B};R\right)\in R^{HW\times H_{G}W_{G}}$ is the embedding position based on the Swin transformer [45]. The features of each head are concatenated and projected by $W_{o}$ to obtain the final output *z*:

(5) $$z=Concat\left(z^{\left(1\right)},\ldots ,z^{\left(M\right)}\right)W_{o}$$

where *M* is the number of multi-head attention mechanism heads and set to 8.

**Figure 4:**
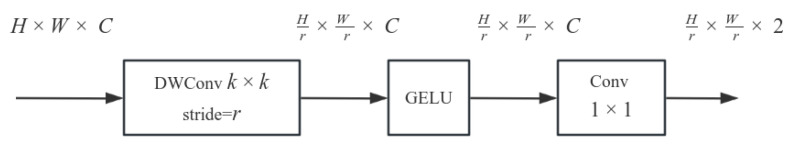
Computation structure of the learned offset $\theta_{offset}$ in deformable convolution. This offset dynamically adjusts sampling positions to better align with signal contours.

The subnetwork structure $\theta_{offset}\left(\cdot \right)$ used to generate the offset is shown in Figure 4. The input features first pass through a deep convolution to capture local features. Then, GELU activation and point-wise convolution are used to generate the 2D offset.

Relative positional bias encodes the relative position between each pair of *q* and *k*, increasing ordinary attention through spatial information. Consider a feature map with a shape of H×W, with relative coordinate shifts in the range of [−H,H] and [−W,W], respectively. Since DAttention has continuous bond positions, the relative displacement in the normalized range is calculated [−1,+1]. The $\varphi (\hat{B};R)$ is then interpolated in the parametric offset table $\hat{B}\in R\left(2H-1\right)\times \left(2W-1\right)$ to override all possible offset values.

### 3.2. Improved Loss Function

Object detection is a critical task in computer vision, where the accuracy of bounding box prediction directly impacts model performance. Intersection over union (IoU) loss functions, such as GIoU and CIoU, have made significant strides in improving detection accuracy by considering factors such as the overlap, distance, and aspect ratio between predicted and ground-truth boxes. However, these methods still face challenges when dealing with complex scenarios, particularly in low-SNR environments. Under such conditions, signal distortion and noise can obscure key features, leading to inaccurate boundary estimation and poor alignment between predicted and true boxes. As a result, these limitations often cause slower convergence and reduced detection accuracy. To address these challenges, this study introduces Focal_SIoU, which integrates the focal mechanism [46] with SCYLLA-IoU (SIoU) [47] to enhance robustness and accuracy, particularly under noisy and distorted signal conditions.

The SIoU loss function, built upon IoU, incorporates additional cost functions such as angle, distance, and shape to more finely optimize the positioning and shape estimation of the target boxes. Its formula is as follows:

(6) $$SIoU=1-IoU+\frac{\Delta +\Omega }{2}$$

Here, IoU is the overlap ratio between the predicted and ground-truth boxes, Δ is the distance cost measuring center misalignment, and Ω is the shape cost evaluating aspect ratio differences. The angle cost Λ is embedded within Δ to modulate sensitivity to directional errors.

The IoU term is defined as the intersection over union between the predicted box *b* and the ground-truth box $b^{gt}$:

(7) $$IoU\left(b,b^{gt}\right)=\frac{Area(b\cap b^{gt})}{Area(b\cup b^{gt})}$$

This value has a range of [0,1], where a higher value indicates greater overlap. However, standard IoU loss does not consider spatial misalignments or shape variation, limiting performance under complex conditions. SIoU addresses this by adding three terms.

The angle cost aims to reduce angular deviation between the center line of the predicted box and the ground-truth box:

(8) $$\Lambda =1-2\times \sin^{2}\left(\arcsin\left(x\right)-\frac{\pi }{4}\right)$$

where

(9) $$x=\frac{C_{h}}{\sigma }=\sin\left(\alpha \right),\;\sigma =\sqrt{{\left(b_{cx}^{gt}-b_{cx}\right)}^{2}+{\left(b_{cy}^{gt}-b_{cy}\right)}^{2}},\;C_{h}=\left|b_{cy}^{gt}-b_{cy}\right|$$

Here, $b_{cx},b_{cy}$ and $b_{cx}^{gt},b_{cy}^{gt}$ are the center coordinates of the predicted and ground-truth boxes. $C_{h}$ is the vertical offset, σ is the Euclidean distance between centers, and α is the angle between them.

The distance cost penalizes the spatial offset between box centers and incorporates the angle factor as follows:

(10) $$\Delta =\sum_{t=x,y}\left(1-e^{-\gamma \rho_{t}}\right),\;\rho_{x}={\left(\frac{b_{cx}^{gt}-b_{cx}}{C_{w}}\right)}^{2},\;\rho_{y}={\left(\frac{b_{cy}^{gt}-b_{cy}}{C_{h}}\right)}^{2}$$

where $C_{w}$ and $C_{h}$ represent the width and height of the smallest enclosing box. γ=2−Λ is an adaptive modulation factor based on the angle difference.

The shape cost accounts for aspect ratio mismatches:

(11) $$\Omega =\sum_{t=w,h}{\left(1-e^{-\omega_{t}}\right)}^{\theta },\;\omega_{w}=\frac{\left|w-w^{gt}\right|}{\max(w,w^{gt})},\;\omega_{h}=\frac{\left|h-h^{gt}\right|}{\max(h,h^{gt})}$$

where w,h and $w^{gt},h^{gt}$ are the width and height of the predicted and true boxes. $\omega_{t}$ reflects their relative difference, and θ (set to 4) controls sensitivity to shape errors.

To further enhance robustness under low-SNR conditions, we incorporate the focal mechanism into SIoU loss. Focal loss increases the weight of difficult samples during training, guiding the model to focus on ambiguous or low-quality predictions:

(12) $$L_{Focal}\left(p_{t}\right)=-\alpha_{t}{\left(1-p_{t}\right)}^{\tau }\log\left(p_{t}\right)$$

Here, $p_{t}$ is the predicted confidence score for the correct class, $\alpha_{t}$ balances class frequencies, and τ controls the degree of focusing on hard examples. A higher τ increases the loss contribution from misclassified or ambiguous samples.

In the object detection task, we compute the SIoU loss for each sample, and we then apply the focal mechanism to re-weigh it as follows:

(13) $$L_{total}=L_{Focal}\left(SIoU(b,b^{gt})\right)$$

This formulation ensures that hard samples—those with a low IoU or large spatial/shape deviations—receive more loss contribution, helping the model to learn to handle them more accurately.

To further illustrate the advantage of Focal_SIoU, we consider two sample cases:

- Box A (easy): IoU=0.85, assume SIoU=0.10;
  $L_{Focal}\left(SIoU\right)=0.10\times {\left(1-0.90\right)}^{2}=0.001$;
- Box B (hard): IoU=0.30, assume SIoU=0.70;
  $L_{Focal}\left(SIoU\right)=0.70\times {\left(1-0.30\right)}^{2}=0.343$.

For the same hard sample (Box B), we compare the computed loss across different loss functions:

- IoU loss: 1−0.30=0.70;
- SIoU loss: 0.70;
- Focal_SIoU loss: 0.343.

Although the absolute loss of Focal_SIoU is numerically smaller than raw SIoU (0.343 vs. 0.70), its key strength lies in emphasizing difficult samples through relative scaling. In SIoU, the loss ratio between hard and easy samples is 0.70/0.10=7, whereas in Focal_SIoU, it is 0.343/0.001=343, amplifying the gradient impact of hard samples by a much larger margin.

This sharp contrast enables the model to ignore easy, well-aligned predictions and focus its learning capacity on challenging, noisy, or misaligned samples. Therefore, Focal_SIoU proves particularly effective in low-SNR environments, where hard examples dominate.

### 3.3. Dataset

In the field of radio sensing, the existing datasets for signal detection have certain limitations. Some researchers have created time–frequency map datasets for simultaneous signal detection and modulation recognition. However, due to the loss of original signal features during the time–frequency transformation process, these datasets only select a few signal modulation types that are easily distinguishable in the time–frequency maps, with SNRs higher than 0 dB, and the signals occupy a larger portion of the time domain in the map. Although these datasets have achieved both signal detection and modulation recognition, there are more convincing datasets available in the field of modulation recognition, which allow researchers to train and test models on a broader range of signal types and under a broader range of SNR conditions [48,49,50]. Furthermore, for the specific task of signal detection, many signals in real-world applications often have SNRs lower than 0 dB, shorter durations, and narrower bandwidths, making them difficult to detect in a broadband environment. This makes it challenging for existing datasets to fully cover these complex scenarios.

To support the proposed broadband signal detection task, we constructed a dataset by processing samples derived from a publicly available modulation classification dataset, HisarMod2019.1 [50]. The original dataset contains 26 modulation types, including common forms, such as AM, FM, PM, FSK, PAM, PSK, and QAM, and five typical wireless channel conditions: ideal, static fading, Rayleigh, Rician, and Nakagami channels. Although the dataset used in this work is synthetically generated, the simulation process strictly adheres to theoretical models of signal modulation and wireless channel propagation. All 26 modulation types are implemented according to their standard waveform characteristics, and channel effects such as additive white Gaussian noise, Rayleigh, Rician, and Nakagami fading are simulated using widely accepted statistical models. These methods ensure that the generated signals are representative of real-world scenarios. Furthermore, the task addressed in this study—detecting signals under broadband and low-SNR conditions—primarily concerns the robustness of time–frequency feature learning. Therefore, using simulated data does not compromise the validity of the experimental results. Nevertheless, applying the proposed method to measured signals is a potential direction for future research.

Based on these narrowband signal samples, we apply several signal processing steps to create broadband detection scenarios. Specifically, each signal is randomly upsampled, time-shifted, frequency-shifted, and injected into a broadband spectrum containing multiple overlapping signals. Additive Gaussian noise and carrier frequency offset are also introduced to simulate low-SNR and frequency misalignment conditions.

**Figure 5:**
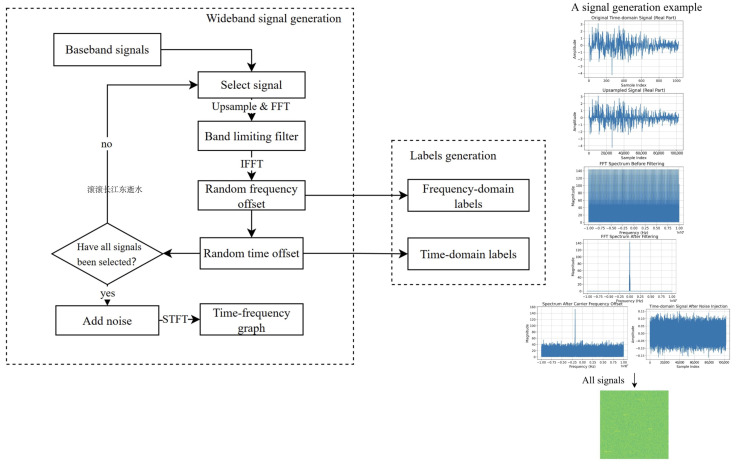
Data generation process for the broadband signal detection dataset. Steps include modulation synthesis using HisarMod2019.1 samples, signal transformation (shifting, upsampling), noise injection, and time–frequency representation.

This processing pipeline results in a large-scale dataset tailored for broadband signal detection, with each sample representing a time–frequency map containing multiple signals and their corresponding labels. Figure 5 illustrates the detailed processing flow and intermediate results. The dataset supports supervised learning for detection models under broadband conditions.

To describe Figure 5, signals under SNR conditions ranging from −8 dB to 6 dB are extracted from the dataset. Based on the needs of this study, specific SNR data are extracted and upsampled. After upsampling, signals are passed through a band-limiting filter and then converted back to the time domain using the Inverse fast Fourier transform (IFFT).

The signals are multiplied by a random frequency offset factor in the form of a complex exponential, and the corresponding frequency offsets are recorded to generate the frequency-domain labels. A random time offset is then added to the signals, and this offset is recorded for generating the time-domain labels. After applying frequency and time offsets, broadband signals are created by iteratively processing all selected baseband signals.

Once all signals have been processed, Gaussian noise is added to the broadband signal to simulate noisy conditions. The noisy broadband signals are then transformed into time–frequency graphs using the short-time Fourier transform (STFT) for network training. The STFT applies a sliding window to observe the signal symmetrically. Fourier transform is performed on the signal within each window, with a window length of 256, a Hamming window type, and a step size of 128. This configuration ensures a good balance between time and frequency resolution, producing smooth time–frequency maps while minimizing spectral distortion.

Table 1 lists the time–frequency map parameters used in the experiments. The time–frequency images are generated with a resolution of 640 × 640 × 3 to align with the input size of mainstream object detection models (e.g., YOLO). This resolution also ensures sufficient granularity to preserve the signal structure in both time and frequency domains. Specifically, using an STFT window of 256 with 50% overlap on a 737,280 sample signal produces 5759 time steps and 129 frequency bins. Reducing the image size below this level would risk losing critical features, particularly in the frequency dimension, where fewer than 129 rows could impair the model’s ability to recognize narrowband signals or weak components under low-SNR conditions. The dataset consists of time–frequency maps containing six signals per image, with SNRs ranging from −8 dB to 6 dB (500 images per SNR level), totaling 4000 images.

**Figure 6:**
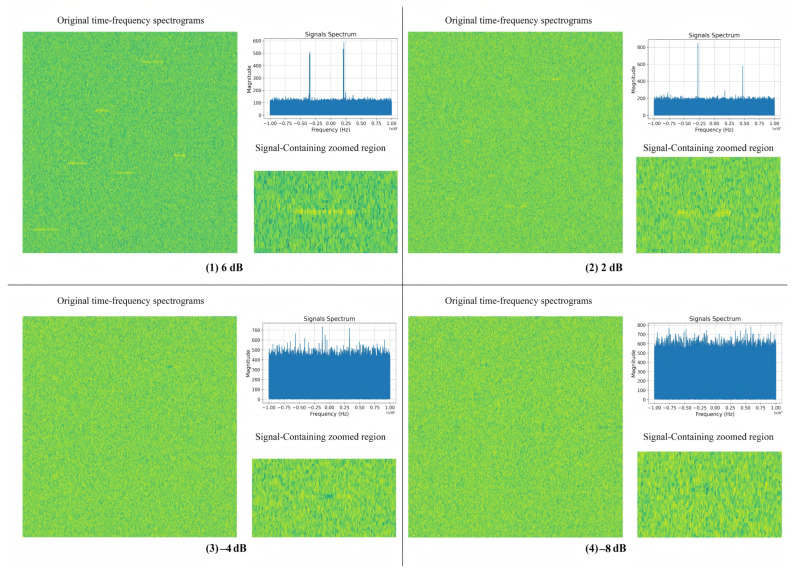
Examples of time–frequency spectrograms and spectral plots at different SNR levels. For each SNR case, Left: full spectrogram; Right: zoomed signal region and frequency-domain. Green denotes lower power, yellow denotes higher power.

As shown in Figure 6, the SNR of the signals in Figure 6 (1–4) decreases from 6 dB to −8 dB. To illustrate the challenges of detecting signals under different noise conditions, Figure 6 presents sample spectrograms and their corresponding frequency-domain representations from the dataset at four representative SNR levels: +6 dB, +2 dB, −4 dB, and −8 dB. For each case, we also provide zoomed-in views of the signal regions to better visualize the degree of interference and signal distortion. These examples highlight how lower SNR levels introduce severe degradation, making signal detection significantly more difficult. The time–frequency locations of the signals are recorded during dataset generation. As the SNR decreases, the signal strength gradually diminishes, making it more difficult to distinguish in the time–frequency map.

## 4. Experiments and Analyses

### 4.1. Experimental Setup and Evaluation Metrics

In all experiments, the random seed was uniformly set to 0 to ensure the reproducibility of the experiments. During training, each batch processed 30 images, and stochastic gradient descent (SGD) was used as the optimizer to update the network parameters. The number of experimental rounds was 2000. To prevent overfitting and improve training efficiency, an early stopping strategy was implemented: if there was no significant improvement in model performance after 50 consecutive training steps (epochs), the training would be automatically stopped. All experiments were conducted on a computer equipped with three NVIDIA RTX 3090 GPUs with 24 GB of VRAM (Dell Inc., Xiamen, China) each. This hardware configuration ensured efficient data processing and computational speed during training, while also enabling highly parallelized and optimized model training and validation. The dataset consisted of time–frequency maps with SNRs ranging from −8 dB to 6 dB, with 500 images per SNR level, for 4000 images. At each SNR level, 20% of the images were used as the validation set, while 80% were used as the training set for model training.

In comparing signal detection results, the mean average precision (mAP), a commonly used metric in machine learning-based object detection, was adopted. mAP takes into account the cases of true positives (TPs), false positives (FPs), and false negatives (FNs) to provide a comprehensive evaluation. The calculation formula used was defined as follows:

(14) $$P\left(k\right)=\frac{TP}{TP+FP}\times 100\%$$

(15) $$R\left(k\right)=\frac{TP}{TP+FN}\times 100\%$$

(16) $$mAP=\frac{1}{C_{s}}\sum_{M=i}^{N}P\left(k\right)\Delta R\left(k\right)$$

where TP is the number of correctly identified signal samples, FP is the number of incorrectly identified or unrecognized signal samples, and FN is the number of incorrectly recognized signal target samples. $C_{s}$ represents the number of signal sample categories, and *M* and *N* represent the number of IoU threshold values. Precision and recall are denoted as P(k) and R(k), respectively.

mAP50 measured the average precision when the intersection over union (IoU) threshold between the predicted box and the ground-truth box was 0.5. mAP50 measured the average precision when the IoU threshold between the predicted box and the ground-truth box was 0.5. mAP50-95 measured the average precision across IoU thresholds ranging from 0.5 to 0.95 in steps of 0.05. A higher mAP50-95 indicated more accurate localization and robustness to prediction errors. These metrics, commonly used in object detection, were adapted here to evaluate the model’s ability to accurately localize signals in the time–frequency domain.

### 4.2. Ablation Study

**Figure 7:**
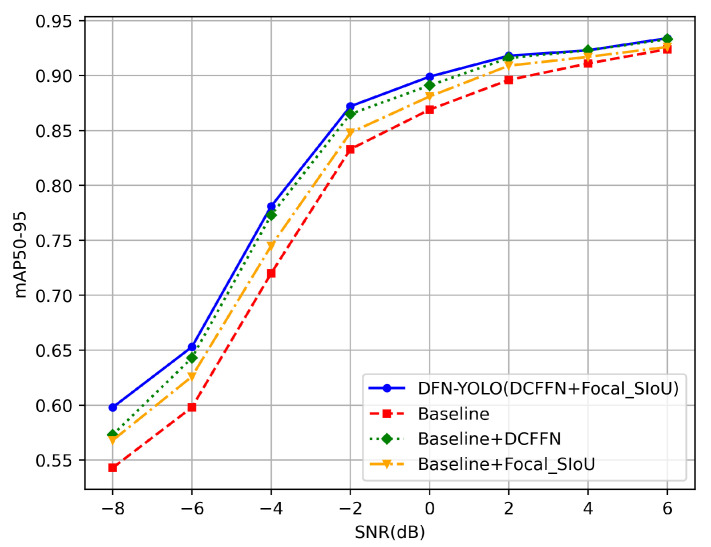
The detection performance of the baseline model and its incrementally enhanced modules under varying SNR conditions.

This section first explores the effectiveness of improving the C2f module to DCFFN and enhancing the loss function. The baseline model used is YOLOv8n, with the experimental results shown in Table 2 and Figure 7.

As shown in Table 2, using DCFFN not only improved the signal detection mAP50-95 from 0.807 to 0.845 but also resulted in a less than 2% increase in the number of parameters. The Focal_SIoU mechanism increased the gradient adjustment weight for difficult samples under low-SNR conditions, thereby improving the accuracy under low-SNR conditions, as well as the overall accuracy. The performance of the network reached its optimal point when both DCFFN and Focal_SIoU were introduced, with mAP50-95 reaching 0.85, proving that both components effectively enhanced the detection accuracy.

Subsequently, based on DCFFN, this section investigates the impact of replacing the C2f module with various SOTA methods on the performance of the baseline model. Specifically, in this ablation experiment, other modular improvements were considered: OREPA, ContextGuided, DLKA, DCNv2-Dynamic, and SCConv. These improvements targeted the baseline model’s C2f structure and aimed to enhance the model’s spatial sensitivity and expressive power by introducing different attention mechanisms or convolution variants. The experimental results are shown in Table 3. The results demonstrate that DCFFN significantly improved model performance while maintaining a relatively low computational cost, highlighting its effectiveness in adjusting the model’s focus to capture richer features.

OREPA [21] used more parameters, reducing the computational load, and achieved slight improvements in both mAP50 and mAP50-95. ContextGuided [25] optimized both parameters and computational cost, but its mAP50-95 was only 0.658, far below the baseline’s 0.807. DCNv2-Dynamic [20,23], with similar parameters and computational load compared to the baseline, increased mAP50 to 0.93, but saw a decrease in mAP50-95, indicating that detection accuracy was sacrificed in favor of higher detection precision. SCConv [24] and DLKA [26] used more parameters and computational resources, showing an improvement in mAP50 compared to the baseline, indicating that these improvements were better at detecting signals when the detection precision requirements were more relaxed. However, their mAP50-95 scores were 0.781 and 0.668, respectively, suggesting that their signal parameter estimations were not sufficiently accurate. In contrast, DCFFN, with relatively low FLOPs and a moderate parameter count, significantly improved both mAP50 and mAP50-95, especially excelling in the more challenging mAP50-95 metric, which reached 0.845.

Next, this section replaced the original C2f module with different stages of DCFFN, and the results are shown in Table 4. From experiments 1 and 2, it can be seen that using the DCFFN structure at earlier stages led to reduced performance gains. Subsequent experiments indicated that using more DCFFN modules at earlier stages led to smaller performance gains. Therefore, it can be concluded that the highest performance gain was achieved when only the C2f at Stage 4 was replaced. Using more DCFFN modules at earlier stages reduced the detection accuracy. Furthermore, using multiple DCFFN modules significantly increased the number of parameters and the computational cost; moreover, with the same batch size, more GPU memory was required during training, which made the hardware requirements less user-friendly.

To further investigate the impact of critical hyperparameters in the DCFFN module, we conducted experiments by varying the number of attention heads (n_heads), the offset range factor, and whether positional encoding (PE) was used. The results are shown in Table 5.

The experimental results demonstrate that increasing the number of attention heads from four to eight improved detection performance. Specifically, mAP50-95 increased from 0.838 to 0.845, representing a relative gain of 0.7%. This improvement came at the cost of a moderate parameter increase from 3.02 M to 3.08 M and a FLOP increase of only 0.1 G, which was considered acceptable.

Regarding the offset range factor, increasing it from 2 to 4 while disabling PE caused a performance drop, with mAP50-95 decreasing from 0.845 to 0.827 (a relative decline of 2.1%), indicating that excessive offset range may harm spatial focus when not properly guided by position information.

Using positional encoding also showed notable benefits. When PE was disabled (with n_heads=8 and offset range = 4), mAP50-95 dropped to 0.827, while enabling PE under the same attention configuration yielded a value of 0.845. This indicated a 2.2% improvement attributable to PE, highlighting its effectiveness in modeling spatial dependencies under deformation.

Overall, the configuration of n_heads=8, offset_range_factor = 2, and use_pe = True achieved the best trade-off between accuracy and computational cost. In scenarios where computational resources were limited, reducing the number of heads to four still maintained relatively high performance (mAP50-95 = 0.838) while reducing the parameter count by approximately 2%.

### 4.3. Parameter Analyses

This section further investigates the impacts of different expected feature map sizes. The size of the expected feature map influences the creation of the position encoding matrix and the grid shape $H_{G}\times W_{G}$.

The results are shown in Table 6. The feature map size of 10 × 5 achieved an mAP50 of 0.929, which is high, only slightly lower than the 5 × 10 size with an mAP50 of 0.931. Compared to other sizes, such as 5 × 5, which achieved an mAP50-95 of 0.927, the performance of 10 × 5 was still superior. The mAP50-95 of 10 × 5 was 0.845, the highest in the table. In comparison to other sizes, such as 5 × 10 with the same area (0.823), 10 × 5 showed a clear advantage. We believe that since signals appear as elongated shapes in the time–frequency map, selecting a non-square expected feature map that better matches its shape would result in better performance. The experimental results confirm this belief: within the appropriate size range, the non-square feature map of 10 × 5 performs optimally. This may be because non-square feature maps have an advantage when detecting narrowband signal blocks in the time–frequency map, allowing for the more accurate capture of these elongated signal features. It is worth noting that, regardless of whether the feature map is square or non-square, this change in size has almost no impact on the model’s complexity.

### 4.4. Performance Comparisons

In this section, DFN-YOLO is compared with SOTA methods, including the YOLOv5 model used in the signal detection method mentioned in Chapter 1 [16], SigdetNet [43], YOLOv9 [51], YOLOv10 [52], and YOLOv11 [53]. To further evaluate the effectiveness of DFN-YOLO, the ED method [5] is also implemented as a traditional baseline, which first filters the original signal into narrower frequency bands to reduce the impact of broadband noise in the time domain.

**Figure 8:**
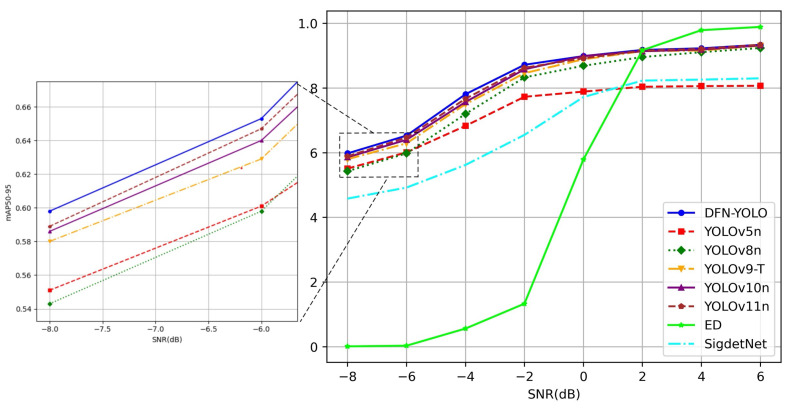
Comparison of DFN-YOLO with other models (YOLOv8, YOLOv9, YOLOv10, YOLOv11, CNN-based, traditional energy detection) in terms of detection accuracy across varying SNR levels.

The experimental results for the SNR range of −8–6 dB are shown in Table 7, and the performance curve of signal detection as a function of SNR is illustrated in Figure 8. As can be seen from Table 7, DFN-YOLO achieves an mAP50-95 of 0.85, outperforming all compared models. Its parameter size (3.08 M) and FLOPs (8.2 G) are slightly higher than YOLOv8n and moderately larger than recent versions such as YOLOv10n and YOLOv11. Compared to SigdetNet and YOLOv5n, DFN-YOLO shows significant improvements in accuracy while keeping the computational burden within an acceptable range.

In terms of inference speed, DFN-YOLO achieves a latency of 1.89 ms, which is only 0.19 ms slower than YOLOv8n (1.7 ms) and faster than YOLOv9-T (2.56 ms), YOLOv10n (1.81 ms), and YOLOv11 (1.75 ms). This indicates that despite the added complexity from DCFFN and deformable attention, the model maintains competitive runtime performance.

In terms of accuracy gain, DFN-YOLO outperforms YOLOv5n by +9.2% (0.850 vs. 0.758), YOLOv8n by +4.3% (0.850 vs. 0.807), YOLOv9-T by +1.9% (0.850 vs. 0.831), YOLOv10n by +1.2% (0.850 vs. 0.838), and YOLOv11 by +0.7% (0.850 vs. 0.843). This demonstrates that DFN-YOLO consistently achieves the best accuracy among all YOLO variants, especially under low-SNR conditions.

The cost of these improvements is modest: Compared with YOLOv8n, DFN-YOLO has only a 2.2% increase in parameters (3.08 M vs. 3.01 M) and the same FLOPs (8.2 G). Compared with YOLOv10n and YOLOv11, its parameter size is slightly higher (by 0.46 M and 0.45 M, respectively), but the detection performance improves by 1.2% and 0.7%.

From a practical deployment perspective, DFN-YOLO’s latency of 1.89 ms per inference corresponds to a throughput of approximately 529 FPS, exceeding the requirement for real-time signal monitoring applications.

Therefore, despite the slight increase in model size and latency, the consistent performance improvement across all metrics indicates that DFN-YOLO achieves a favorable trade-off between detection accuracy and computational cost, supporting its real-world applicability in low-SNR signal detection tasks.

In Figure 8, it can be observed that when the SNR reaches 0 dB or higher, the mAP50-95 exceeds 0.9. As the SNR increases to 6 dB, the mAP50-95 improvement is only 0.03. At this point, the performance of DFN-YOLO is close to those of YOLOv9 to YOLOv11, but it still maintains optimal performance. When the SNR is below 0 dB, using the Focal_SIoU function in the proposed method allows it to focus more effectively on handling low-quality and more challenging data under low-SNR conditions, maintaining a high detection accuracy. As a result, even at −8 dB, the mAP50-95 still reaches 0.6, and at −4 dB and −2 dB, the proposed method significantly outperforms the other models. While the ED method achieves an mAP50-95 of only 0.133 at −2 dB and almost zero at SNRs below −2 dB, DFN-YOLO maintains robust performance, with an mAP50-95 score reaching 0.6 at −8 dB. Moreover, although the ED method performs better at high SNRs (above 4 dB), its inference time is much longer (6.57 s per signal image), making it less suitable for real-time or large-scale signal detection tasks. In contrast, DFN-YOLO demonstrates a favorable balance between accuracy and computational efficiency, especially under challenging conditions such as low-SNR and broadband scenarios, which are the focus of this study.

When the model achieves optimal performance, evaluating its accuracy in terms of start and stop time estimation, as well as center frequency estimation, becomes crucial. This not only helps to validate the overall performance of the model but also provides the essential foundation for subsequent signal separation and processing tasks. In this section, we conduct a detailed analysis of the estimation errors for signal parameters, including start–stop time errors and center frequency errors. These analysis results will help us to gain a deeper understanding of the model’s precision and provide valuable data to support further optimization and improvement. The data in Table 8 report the parameter estimation errors under the condition that the signal detection IoU is greater than 50.

According to the analysis in Table 8, there is no significant difference in center frequency estimation errors across models. Relative to the 20 MHz frequency range of the time–frequency plot, the maximum average relative error is 0.14%. As the SNR increases, all models show higher accuracy in estimating the signal’s start time, end time, and center frequency. A horizontal comparison reveals that DFN-YOLO already achieves a high level of start–stop time estimation accuracy at −8 dB, with the average error reduced by at least $3\times 10^{-5}$ s compared to other models. Considering that the time range of the time–frequency plot is 0.04 s, the start and end time errors of the proposed method at −8 dB only account for 0.1% of the entire time–frequency plot.

Combining the data from Table 7 and Table 8, DFN-YOLO performs the best, followed by YOLOv11n, with YOLOv10n and YOLOv9-T slightly lagging behind, while YOLOv5 shows a significant performance gap compared to the other models. This indicates strong consistency between the signal parameter estimation errors and the signal detection performance of each model.

Notably, although the ED method achieves correct detections under certain SNR conditions, its parameter estimation accuracy is still inferior to that of DFN-YOLO. However, the ED method performs slightly better in center frequency estimation in the cases that it detects successfully. This is because it first applies narrowband filtering, which inherently restricts the possible estimation error range in the frequency domain. Therefore, the detected signals are constrained within a narrower band, leading to smaller estimation deviation. At −8 dB, ED fails to detect any signals, so it is excluded from comparison under this condition.

Since the signals in the dataset have a wide bandwidth, slight shifts in the detection box may lead to large errors in center frequency estimation. However, methods such as frequency shifting, low-pass filtering, and down-sampling can be used to make more accurate frequency estimates for narrowband signals. DFN-YOLO can accurately estimate the time, crop the signal in the time domain, and provide an initial estimate of the signal’s center frequency, sufficient to support subsequent signal separation and precise frequency estimation tasks.

**Figure 9:**
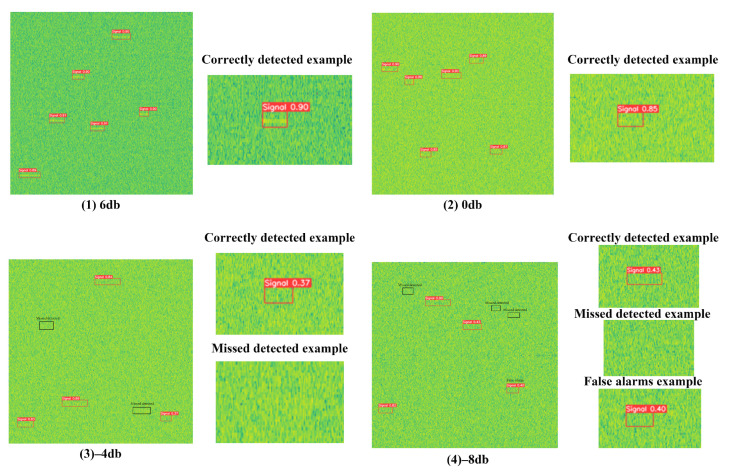
The detection results of the proposed method for time–frequency spectrograms under various SNR conditions (6 dB to −8 dB). Enlarged views of representative cases—correct detection, false alarm, and missed detection—are provided to highlight detection performance under different noise conditions.

To provide a quantitative perspective, we further count the detection performance of DFN-YOLO in the four representative examples of Figure 9. To better illustrate the impact of decreasing SNR on detection performance, four representative SNR levels are chosen: +6 dB as a high-quality baseline, and 0 dB to −8 dB to demonstrate the performance degradation under progressively noisier conditions. At +6 dB, all six signals are correctly detected with no false alarms or missed detections. At 0 dB, six signals are correctly detected, with no false alarms and missed detections. At −4 dB, four signals are correctly detected, two are missed, and zero false alarms occur. At −8 dB, three signals are correctly detected, while three are missed and one false alarm is observed. These results clearly reflect the impact of the SNR on detection performance and are consistent with the statistical results in Table 8.

To evaluate the performance of DFN-YOLO under extremely low-SNR conditions not included in the training range, we tested the model trained with SNRs from −8 dB to +6 dB on two additional test sets at −10 dB and −12 dB. As shown in Table 9, the model still achieved acceptable detection performance, with mAP50 scores of 0.402 at −10 dB and 0.445 at −12 dB, demonstrating strong generalization capability. Moreover, after retraining the model with additional −10 dB and −12 dB data, only marginal improvements were observed (e.g., mAP50 increased to 0.497 at −10 dB and 0.525 at −12 dB), further confirming the robustness of the proposed method.

To further validate robustness, a new model trained using −12 dB to +6 dB data was also evaluated on the same test sets. The results demonstrate similar performance, with only marginal improvement, showing that DFN-YOLO is robust to variations in noise levels. Compared with other YOLO variants and the traditional ED method, DFN-YOLO maintains significantly better detection accuracy and lower false alarm rates at extremely low SNRs.

These results highlight the practical potential of the proposed method in challenging wireless sensing scenarios.

### 4.5. Key Findings Summary

Based on the comprehensive experimental analyses above, the following key findings are highlighted:

- Effectiveness of DCFFN:The proposed DCFFN module significantly improves detection performance, especially on the stricter mAP50-95 metric, with a minimal increase in parameters and computational cost.
- Robust Loss Function: The Focal_SIoU loss enhances detection accuracy under low-SNR conditions by assigning higher gradients to difficult samples, making the model more robust in challenging environments.
- Module Placement Strategy: Replacing only the Stage 4 C2f with a DCFFN yields the highest accuracy and best trade-off between performance and resource cost, avoiding redundancy.
- Offsets Mechanism: The inclusion of the offset mechanism contributes to significant accuracy gains with negligible additional cost, confirming its necessity in the design of DCFFNs.
- Feature Map Size: Non-square feature maps (e.g., 10 × 5) better match signal shapes in the time–frequency map and lead to superior detection results.
- Comparison with SOTA Methods: DFN-YOLO outperforms other YOLO series models and traditional methods (like ED) in terms of detection precision, parameter estimation accuracy, and speed, particularly under low-SNR conditions.
- Generalization Capability: Even when tested on SNR levels lower than the training range (−10 dB and −12 dB), DFN-YOLO maintains strong performance, demonstrating excellent generalization and practical applicability.

## 5. Conclusions

This study explores potential improvements to the object detection model for blind signal detection in broadband time–frequency plots. For small-target signal detection tasks, we optimized the original model and constructed DFN-YOLO. By dynamically adjusting the attention focus during channel feature fusion, the model enhanced its ability to capture narrowband signals in broadband time–frequency plots, playing a crucial role in improving the detection accuracy of small-target signals. Additionally, we introduced the Focal_SIoU function, which increased the detection accuracy for high-difficulty samples under low-SNR conditions.

Furthermore, this study describes the methodology for constructing a signal detection dataset and validates the effectiveness of the proposed improvements through a series of ablation experiments. The experimental results not only demonstrate the superior performance of the improved DFN-YOLO but also show a comparison with mainstream object detection models, highlighting performance improvements. The average mAP50-95 achieved was 0.850. The average error in time estimation for signals was within $5.55\times 10^{-5}$ s.

Future studies in this field may focus on the following:

1. Use more real-world measured signals to enrich the dataset with additional signal types, modulation schemes, and transmission parameters (e.g., symbol rate).
2. Implement lightweight network modifications to facilitate deployment in practical applications.
3. Introduce unsupervised learning techniques or zero-shot methods (e.g., YOLO-World) to enhance model adaptability for handling unlabeled or sparsely labeled data.

## Figures and Tables

**Table 1:** Time–frequency map parameters and dataset characteristics.

Parameter	Description
Time Frame Duration	0.04 s
Frequency Range	−10–10 MHz
Sampling Rate	20 MHz
Spectrogram Size	640 × 640 × 3 (RGB image)
Signal Duration	0.0025–0.005 s
SNR Range	−8–6 dB
Total Samples	4000 spectrograms × 6 variants = 24,000 samples
Modulation Types (26)	AM–DSB, AM–SC, AM–USB, AM–LSB, FM, PM, 2–FSK, 4–FSK, 8–FSK, 16–FSK, 4–PAM, 8–PAM, 16–PAM, BPSK, QPSK, 8–PSK, 16–PSK, 32–PSK, 64–PSK, 4–QAM, 8–QAM, 16–QAM, 32–QAM, 64–QAM, 128–QAM, 256–QAM
Channel Models	Ideal, Static Fading, Rayleigh, Rician, Nakagami

**Table 2:** The DFN-YOLO ablation experiments.

	Methods	mAP50	mAP50-95	Parameters	FLOPs (G)	Inference Time (ms)
1	Baseline	0.917	0.807	3.01 M	8.2	1.70
2	Baseline+DCFFN	0.929	0.845	3.08 M	8.2	1.89
3	Baseline+Focal_SIoU	0.922	0.816	3.01 M	8.2	1.72
4	DFN-YOLO (DCFFN+Focal_SIoU)	0.931	0.850	3.08 M	8.2	1.89

**Table 3:** Ablation experiments with the C2f modification methods.

	Methods	mAP50	mAP50-95	Parameters	FLOPs (G)	Inference Time (ms)
1	Baseline	0.917	0.807	3.01 M	8.2	1.70
2	Baseline+DCFFN	0.929	0.845	3.08 M	8.2	1.89
3	Baseline+OREPA	0.923	0.812	3.72 M	8.0	2.05
4	Baseline+ContextGuided	0.915	0.658	2.73 M	8.0	1.76
5	Baseline+DLKA	0.924	0.668	3.66 M	8.7	2.17
6	Baseline+DCNv2-Dynamic	0.930	0.787	3.05 M	8.1	2.01
7	Baseline+SCConv	0.924	0.781	3.31 M	8.7	2.11

**Table 4:** Ablation experiments with DCFFN applied at different stages.

Stage 1	Stage 2	Stage 3	Stage 4	mAP50	mAP50-95	Parameters	FLOPs (G)	Inference Time (ms)
0	0	0	1	0.929	0.845	3.08 M	8.2	1.89
0	0	1	0	0.916	0.802	3.06 M	8.3	1.92
0	0	1	1	0.915	0.665	3.11 M	8.4	2.10
0	1	1	1	0.913	0.653	3.12 M	8.5	2.65
1	1	1	1	0.912	0.626	3.27 M	9.2	3.22
Baseline				0.917	0.807	3.01 M	8.2	1.70

**Table 5:** Performance of DCFFN with different hyperparameter settings.

n_heads	Offset Range Factor	Use PE	mAP50	mAP50-95	Params (M)	FLOPs (G)	Inference Time (ms)
4	2	True	0.925	0.838	3.02	8.1	1.84
8	2	True	0.929	0.845	3.08	8.2	1.89
8	4	False	0.921	0.827	3.08	8.2	1.96
8	0	True	0.913	0.812	3.08	8.2	1.88
4	4	True	0.922	0.832	3.02	8.1	1.91

**Table 6:** An exploration of the size parameters of the expected feature map in the DCFFN module.

	Size	mAP50	mAP50-95	Parameters	FLOPs (G)	Inference Time (ms)
1	5 × 5	0.927	0.837	3,078,819	8.2	1.9
2	10 × 5	0.929	0.845	3,078,819	8.2	1.89
3	5 × 10	0.931	0.823	3,078,819	8.2	1.89
4	10 × 10	0.922	0.813	3,078,819	8.2	1.88
5	15 × 15	0.927	0.813	3,078,819	8.2	1.88
6	20 × 10	0.925	0.833	3,078,819	8.2	1.89
7	10 × 20	0.929	0.830	3,078,819	8.2	1.88
8	20 × 20	0.926	0.843	3,078,819	8.2	1.87
9	10 × 40	0.922	0.819	3,078,819	8.2	1.88
10	40 × 10	0.927	0.829	3,078,819	8.2	1.88
11	25 × 25	0.919	0.807	3,078,819	8.2	1.87
12	30 × 30	0.926	0.838	3,078,819	8.2	1.87

**Table 7:** Performance comparison of DFN-YOLO and other models. ’XX’ means the metric is not reported for ED.

	Methods	mAP50-95	Parameters	FLOPs (G)	Inference Time (ms)
1	ED	0.457	XX	XX	6467
2	SigdetNet	0.656	362,412	0.34	0.9
3	YOLOv5n	0.758	1,924,586	4.5	1.43
4	YOLOv8n	0.807	3,011,043	8.2	1.7
5	YOLOv9-T	0.831	2,034,256	7.7	2.56
6	YOLOv10n	0.838	2,318,169	6.7	1.81
7	YOLOv11	0.843	2,636,545	6.5	1.75
8	DFN-YOLO	0.850	3,078,819	8.2	1.89

**Table 8:** Different models detect the signal parameter estimation error. ’XX’ means the metric is not reported for ED.

Methods	Index	−8 db	0 db	6 db
DFN-YOLO	Mean start time error (s)	$5.27\times 10^{-5}$	$4.06\times 10^{-5}$	$4.14\times 10^{-5}$
	Mean end time error (s)	$5.55\times 10^{-5}$	$4.04\times 10^{-5}$	$4.17\times 10^{-5}$
	Mean center frequency error (Hz)	24,644.61538	7629.5	6716.5
	Mean start time error (s)	$6.05\times 10^{-5}$	$5.02\times 10^{-5}$	$4.32\times 10^{-5}$
YOLOv11n	Mean end time error (s)	$6.34\times 10^{-5}$	$6.83\times 10^{-5}$	$4.68\times 10^{-5}$
	Mean center frequency error (Hz)	26,015.38462	7148.5	6291
	Mean start time error (s)	$8.08\times 10^{-5}$	$7.11\times 10^{-5}$	$5.51\times 10^{-5}$
YOLOv10n	Mean end time error (s)	$7.58\times 10^{-5}$	$8.83\times 10^{-5}$	$5.68\times 10^{-5}$
	Mean center frequency error (Hz)	28,015.38462	8008.5	6481
	Mean start time error (s)	$8.35\times 10^{-5}$	$8.01\times 10^{-5}$	$6.28\times 10^{-5}$
YOLOv9-T	Mean end time error (s)	$7.34\times 10^{-5}$	$7.85\times 10^{-5}$	$6.54\times 10^{-5}$
	Mean center frequency error (Hz)	27,563.45798	7324.5	6436
	Mean start time error (s)	$1.36\times 10^{-4}$	$1.08\times 10^{-4}$	$9.19\times 10^{-5}$
YOLOv8n	Mean end time error (s)	$7.35\times 10^{-5}$	$9.79\times 10^{-5}$	$7.59\times 10^{-5}$
	Mean center frequency error (Hz)	27,263.07692	7237.5	6366
	Mean start time error (s)	$2.17\times 10^{-4}$	$1.87\times 10^{-4}$	$1.26\times 10^{-4}$
YOLOv5n	Mean end time error (s)	$1.63\times 10^{-4}$	$2.06\times 10^{-4}$	$1.40\times 10^{-4}$
	Mean center frequency error (Hz)	24,632.85714	8745	7095
	Mean start time error (s)	$2.47\times 10^{-4}$	$1.80\times 10^{-4}$	$1.16\times 10^{-4}$
SigdetNet	Mean end time error (s)	$1.92\times 10^{-4}$	$1.96\times 10^{-4}$	$1.20\times 10^{-4}$
	Mean center frequency error (Hz)	25,283.35158	8458	6892
	Mean start time error (s)	XX	$6.65\times 10^{-4}$	$1.53\times 10^{-4}$
ED	Mean end time error (s)	XX	$2.06\times 10^{-4}$	$1.67\times 10^{-4}$
	Mean center frequency error (Hz)	XX	5323	4212

**Table 9:** Comparison of detection performance under extremely low-SNR conditions.

SNR	Model (Training Range)	mAP50	mAP50-95
−12 dB	DFN-YOLO (−8 dB to +6 dB)	0.445	0.25
	DFN-YOLO **(−12 dB to +6 dB)**	**0.525**	**0.275**
	YOLOv11n (−8 dB to +6 dB)	0.438	0.242
	YOLOv10n (−8 dB to +6 dB)	0.426	0.238
	YOLOv9n (−8 dB to +6 dB)	0.421	0.233
	YOLOv8n (−8 dB to +6 dB)	0.405	0.226
	YOLOv5n (−8 dB to +6 dB)	0.408	0.185
	SigdetNet (−8 dB to +6 dB)	0.368	0.178
	ED	0.001	0
−10 dB	DFN-YOLO (−8 dB to +6 dB)	0.402	0.228
	DFN-YOLO **(−12 dB to +6 dB)**	**0.497**	**0.26**
	YOLOv11n (−8 dB to +6 dB)	0.385	0.22
	YOLOv10n (−8 dB to +6 dB)	0.382	0.216
	YOLOv9n (−8 dB to +6 dB)	0.375	0.211
	YOLOv8n (−8 dB to +6 dB)	0.368	0.201
	YOLOv5n (−8 dB to +6 dB)	0.386	0.176
	SigdetNet (−8 dB to +6 dB)	0.389	0.172
	ED	0.001	0

## Data Availability

The training dataset, trained models, programs for data generation, training results, and logs will be prepared by the authors and publicly shared at https://github.com/pprrkkxx/dfn-db, accessed on 5 July 2025.

## Abbreviations

The following abbreviations are used in this manuscript:

ED	Energy Detection
SNR	Signal-to-Noise Ratio
YOLO	You Only Look Once
FRCNN	Faster Region Convolutional Neural Network
DCN	Deformable Convolutional Network
OREPA	Online Convolutional Reparameterization
DAttention	Deformable Attention
DLKA	Deformable Large-Kernel Attention
DFN-YOLO	Deformable Feature-Enhanced Network–You Only Look Once
DCFFN	Deformable Channel Feature Fusion Network
C2f	Concatenate to Fusion
Focal_SIoU	Focal Scaled Intersection over Union
mAP50-95	Mean average precision averaged over IoU thresholds ranging from 0.50 to 0.95
SIoU	SCYLLA-IoU
